# Patients' Experiences in Inviting Healthcare Persons Into Caring Relationships: A Thematic Analysis

**DOI:** 10.1111/scs.70254

**Published:** 2026-04-19

**Authors:** Marja Leena Portaankorva, Anne Kasén, Lisbeth Fagerström, Heli Vaartio‐Rajalin

**Affiliations:** ^1^ Faculty of Science and Engineering, Department of Nature and Health Sciences Åbo Akademi University Vaasa Finland; ^2^ Inflammation Center Helsinki University Hospital Helsinki Finland; ^3^ New Children's Hospital Helsinki Finland; ^4^ Faculty of Nursing and Health Sciences Nord University Bodø Norway; ^5^ Turku University of Applied Sciences Turku Finland

**Keywords:** caring relationship, invitation, suffering, thematic analysis

## Abstract

**Aim:**

This study explored patients' experiences in inviting healthcare persons into the caring relationship.

**Introduction:**

Healthcare persons (i.e., physicians and nurses in this study) are usually responsible for inviting patients into caring relationships. Limited research has been published about patients who actively initiate relationships with healthcare persons, and their invitations are sometimes overlooked.

**Method:**

A qualitative approach was applied. Data were collected using semi‐structured interviews with 20 adult patients in outpatient care between January and April 2023 and analysed with inductive thematic analysis.

**Findings:**

Patients initially invited healthcare persons into the caring relationships. The themes are (1) patients tried to contact healthcare persons in a human‐to‐human way with polite small talk and discussion; (2) patients advocated for themselves and insisted on being heard if their suffering was not seen and understood; (3) patients strongly expressed their suffering and deep worries about wanting to be seen and understood and (4) patients experienced having their suffering seen and understood.

**Conclusion:**

Patients' invitation can be interpreted as a patient's attempt to bring healthcare persons to a closer understanding of patients' suffering. Patients' invitation activity levels with healthcare persons differed based on how their first invitations were attended to or heard. Hearing a patient's invitation is an ethical challenge to practicing healthcare persons because even silent invitations should be heard, and more research is needed concerning healthcare persons in this area. More research is also needed on patients' invitations in different life and health situations; research is equally needed about patients' experiences of healthcare persons' invitations and how healthcare persons' invitations influence patients' dignity.

## 1 Introduction

Invitations in health care, such as those for preventive screenings, follow‐up appointments after a patient's initial contact regarding a health problem, are generally perceived as initiated by the organisations. However, a patient's contact can also be understood as a call for or an invitation to a caring relationship. The hectic schedules and efficiency requirements for healthcare persons today may lead to overlooking this invitation and the patient's unique needs. But what do we really know about patients' experiences of invitation in health care?

Registered nurses' (RNs') invitations emerge in the literature research of clinical communication where RNs' and patients' different communication styles constructed a critical starting point for interaction in the care [1]. For example, patient‐centred communication contained RNs' invitation to the patients which encouraged the patients to share their experiences and participate in their care, and the interpersonal interaction became symmetrical. On the contrary, RNs' dominant discourse of communication was described in non‐patient‐centred care [1]. RNs' invitations to the patients dwell in the patient‐centred communication [1, 2] that had positive effects to patients' engagement [3], satisfaction [3, 4] and safety [5], and it might relieve racial and cultural differences between healthcare persons and patients [4].

The patients' communication is sparsely researched, and patients' invitation might appear as taking the initiative to talk about emotional concerns and using non‐verbal strategies, such as humour [1]. A need to recognise and interpret patients' emotional expressions is identified, and Verona Coding Definitions of Emotional Sequences (VR‐CoDES) has been used in analysing healthcare persons' responses to patients' emotional expressions which may appear as vague cues or clear concerns [6]. These vague cues can be interpreted as patients' indefinite invitations. Healthcare persons should recognise patients' expressions and provide positive explicit responses and space for further emotions [6]. In turn, using VR‐CoDES, patients preferred responses to their expressed emotions that demonstrated clinicians' engagement, but empathetic statements were not so significant [7].

A patient's invitation is given an official and articulated position in the SPIKES protocol (Setting, Perception, Invitation, Knowledge, Empathy, Summary) [8]. A patient's invitation means the physician receives a patient's permission to communicate bad news concerning a treatment failure [8], and the patient is ready for information about their disease [9]. A patient's invitation triggers a physician's response to share information [10].

Patients as inviting participants in the caring relationship emerged in early rehabilitation in the critical care environment where patients invited nurses by spontaneous movements during caring procedures. However, nurses did not always notice or respond to these invitations [11]. Petticrew [12] found that a patient's invitation contained characteristics of voluntariness and vulnerability that allowed the nurse to face a patient's sensitive presence as another human. However, nurses' hiding in a professional role or turning away might happen, too [12]. Patients invited the nurse into the caring relationship not only because they wanted answers to their questions, to discuss health matters, and create a relationship with the nurse to share their experiences of suffering but also because they wanted the nurse to do something important to them (e.g., a small favour) [13].

According to earlier research, RNs' invitation is possible to find in the patient‐centred communication [1, 2]. Patients' invitation is identifiable in patients' emotional expressions [6] and patients' invitations might encourage professionals engaging in caring relationships [7], and sharing knowledge [8, 9, 10] and presence [12, 13]. Knowledge of the patients' invitations and their experiences of these invitations seems sparse and scattered in occasional appearances in the caring and nursing research. Therefore, this study explores how patients describe their invitations to healthcare persons to better identify invitations' characteristic features and improve responsiveness.

### 1.1 The Theoretical Framework of Invitation

Eriksson's theory of human suffering [14] highlights the importance of a nurse's invitation to a patient. For example, by indicating that not being welcomed is a form of suffering for the patient. Suffering insults a human being's dignity and causes shame. An invitation is the opposite of being unwelcome and confirms human dignity [14] that is the most important purpose of caring and nursing ethics [15].

Eriksson's theory of caritative caring ethics [16] describes both a patient's and healthcare person's invitations as reciprocal in caring encounters [17]. A patient's invitation is characterised as an appeal for charity that a nurse should meet in a caring communion in every encounter [16], affirming that the patient is always welcomed to the caring relationship for rest and genuine hospitality [18]. A patient's invitation exists as a continuous appeal to a nurse to become invited, and patients invite healthcare persons because they have concerns about their health and illness [19]. Patients' expressions of health problems, needs and desires embody a message of suffering to the nurse. Patients also pondered their confidence in the nurses' competence, experiences of comfort, guidance, discussions and closeness [20]. A patient's message of suffering to the nurse can also be understood as a part of that patient's invitation.

According to Purnell et al. [21], a patient's invitation is a patient's call for nursing that an authentically present nurse hears in a unique nursing situation. The nurse enters the patient's world, into the space of ‘caring between’, where constant and mutual unfolding enhance the loving relation. Both a nurse and a patient are caring persons. Living caring is a lifelong process in which people learn what it means to nurture each other as caring and to grow caring in mutual respect [21, 22].

A patient's invitation can also be viewed through the lens of the philosopher Lévinas [23], who considered how human relations are constructed. When a healthcare person and a patient have an encounter, their faces affect each other. The face—one's presentation of oneself—is naked, poor and unfamiliar, yet holy and high, appealing to one's authorisation, one's powers; it opposes me and speaks to me to do no harm and commit no violence. The face invites me into a relationship where the other teaches me something about myself [23], and I serve or offer myself unconditionally to the other without the ability to free myself from responsibilities towards the other [24]. The relationship is asymmetric from both parties' viewpoints because both experience having more responsibility than the other and feel the need to invite first [25].

To summarise, invitations by healthcare persons and patients have not gained much attention in nursing research. However, in caring theories, such as those of Eriksson and Boykin and Schoenhofer, as well as in Levinas's philosophy, invitation is discussed as a deeply ethical phenomenon.

### 1.2 Aim

This study aimed to explore patients' experiences in inviting healthcare personnel into the caring relationships.

## 2 Method

The study's approach was qualitative. Data were collected in individual interviews [26] and analysed using inductive thematic analysis according to Braun and Clarke [27] because this method does justice to the patients' experiences of qualitative phenomena without separating those experiences from their context.

### 2.1 Setting and Participants

Patients, who had extensive experiences with various types of relationships with healthcare persons, were searched for as participants. Day hospitals in outpatient clinics were included as context for data collection. Patients with severe, long‐term, or myriad illnesses requiring continuous care are mostly treated in hospital outpatient clinics in Finland, and the patients probably have the energy to participate. Three nursing directors at a university hospital in Finland received e‐mails about this study; two introduced the topic to their head nurses, two of whom invited the first author to tell them and the other nurses about the study in unit meetings.

The participant inclusion criteria were over age 18 and having had multiple experiences of relationships with healthcare persons, as well as the interest, willingness and capability to discuss those encounters and about building those caring relationships.

Purposeful sampling was utilised [28]. The registered nurses who recruited the participants were given a short summary of the research information by explaining the study's aim, inclusion criteria and procedure to the participants. The summary of research information was kept available in the day hospital waiting room. Based on this information, one man and one woman volunteered to participate, and the nurses succeeded in recruiting six men and 12 women, totaling 20 patients. The participants were between 35 and 83 years old. The guiding principle of meaning saturation was followed for estimating the number of participants for interviews in the qualitative study [29]. The meaning saturation was achieved after interviewing 20 participants.

### 2.2 Data Collection

Data collection was conducted with individual interviews [26]. These enabled conversations that made a deeper level understanding possible—patients' intentions and explanations—through additional questions. The researcher group created an interview guide of semi‐structured questions: It was first tested informally with the first author's friends, then some of the text was reworded, some unnecessary questions were deleted, and others were rewritten to focus better on the aim. Table 1 presents the interview guide. The concept of ‘initiative in creating a caring relationship’ was helpful in the interviews and was used along with the concept of ‘to invite into the caring relationship’ to express how a patient decides to call a healthcare person without awaiting a healthcare person's invitation.

**TABLE 1:** Interview guide.

In which ways you have invited the health care personnel (nurses and physicians) to help you? How have you been inviting/initiative in the care? How have you created your caring relationships in the care? What kind of successions did you have when you were inviting/initiative in the care? What kind of consequences there were when you were inviting/initiative? Can you reflect how your self‐respect influence on your invitation in the care and how your openly presented invitation influences on your self‐respect? How has health care personnel shown to recognise your initiative or invitation? How is your invitation and initiative accepted in the care? How patient achieves a kind encounter with health care personnel? To what extent do you think you can influence on how welcomed you are in the care?

The first author was a female RN with a master's degree in caring science and many years of experience in inpatient and outpatient care, and unknown to participants. She conducted the interviews in Finnish, between January and April 2023 in the day hospital. One participant gave a telephone interview. Some were interviewed in a separate room; others in a bigger room with other patients but at a distance that provided the privacy to talk freely. The nurses came to the rooms several times during the interviews, so the interviews were sometimes interrupted for some minutes. The interviews proceeded dialogically: the interviewer could ask clarifying questions and answer the participants' questions.

The interviews were recorded and varied between 20 and 160 min; the median was 53 min. The interviewer made additional notes of the participants' questions and gestures. A senior member of the research group checked the first three transcribed interviews and gave instructions for the next interviews.

### 2.3 Data Analysis

The transcribed text was 180 A4 pages in 11‐point font and single‐line spacing. The shortest section was three pages, and the longest was 24; the median was nine.

According to Braun and Clarke [27], a thematic analysis can be used inductively or deductively; this study applied the inductive modality. Meaning codes and themes were derived from the text because no theory exists of a patient's invitation from which researchers could select the coded categories [30]. Reflexivity in thematic analysis underlines the researcher's subjectivity, reflexive engagement with theory, text and interpretation, and the fact that knowledge is contextual. Additionally, the reflexivity involved that the researchers iteratively developed the codes and the themes until consensus was reached.

The thematic analysis comprised six steps [31]. First, we familiarised ourselves with the text by transcribing, reading and re‐reading, and taking notes of the possible initial codes and themes. The study's interviews were transcribed immediately after being recorded so the first initial themes could be outlined. Second, the first author created the initial codes from the intriguing features of the text and gathered the relevant text pieces. This phase was performed after all the text was transcribed. Third, we searched jointly for the themes by comparing the initial codes with the potential themes. Fourth, we reviewed the themes by checking how they served in relation to the coded text extracts and the whole text. Fifth, we defined and named the themes. Sixth, we wrote the report according to the thematic analysis reporting guidelines [31] and to the consolidated criteria for reporting qualitative research (COREQ) instructions [32]. Table 2 presents an example of creating a theme.

**TABLE 2:** An example of creating one of the themes from the codes through the sub‐themes.

Text quotations	Codes from the text	Sub‐themes	Theme
*I feel that I am quite actively in connection, this care is relatively passive to me, I meet the doctor once a year, so without my own initiative I would be quite alone*	I am quite active	Taking initiative and making contact	1 Patients tried to contact healthcare persons in a human‐to‐human way with polite small talk and discussion
	To be in connection		
	My own initiative means not to be alone		
*… and now you must [be initiative]… it is I who calls the nurse … I know I'll get help when I need, I get the connection for discussion*	You must be initiative		
	It is I who calls the nurse		
*Well, here in the day‐hospital I greet when I come and I tell why I am here before they ask and so* …	I greet when I come	Greeting, talking and discussing in a friendly way	
	I tell why I am here before they ask		
*If I talk much they easily answer to me and in that way I am easier in contact than another kind of person who doesn't talk and he may just follow up, and that's it*	If I talk much … I am easier in contact		
	If I talk much … they answer to me		
*I think and weigh, can I ask it. Probably I have been scanning, how busy they seem and how often they come here and do they come spontaneously or do the walk by and check or do they cone near here*	I think and weigh can I ask	Exploring the atmosphere for proposing things in a right way	
	I have been scanning their haste		
	I have been scanning do they come near		
*Well, the [hospital] game means that you must present things in a right way, you must be able to ask and say things in a right way* … *and in some cases it is useful. My friend has dyslexia* … *she becomes misunderstood often. We together practice and think how to answer to the email and what is best to say in the telephone. It is hard but necessary for a person with many diseases*	Present things in a right way		
	Ask in a right way		

### 2.4 Ethical Considerations

The research permit (HUS/218/2022) was obtained in October 2022. HUS Ethics Committee gave the statement (HUS/13407/2022). All study procedures were performed according to ethical research regulations [33]. The research information and consent form was created according to the hospital research solicitors' instructions. Participants were informed in written form, then orally by the recruiting nurses who had an official caring relationship with the patients, after which the interviewer introduced herself and informed the participants about the study's aim and the procedure, and about that the participation was voluntary, interruptible and non‐care‐affecting; they signed one informed consent for themselves and one for the researcher. Data was saved on a password‐protected digital environment.

## 3 Findings

A patient's invitation involved many ways of seeking contact, connection and relationships with healthcare persons. Findings consisted of four main themes that are presented with the categories and sub‐categories in Table 3. The findings are opened through their text extracts in what follows. The sub‐themes and text extracts are *italicised*.

**TABLE 3:** Findings with main themes and sub‐themes.

Main themes	1 Patients tried to contact healthcare persons in a human‐to‐human way with polite small talk and discussion	2 Patients advocated for themselves and insisted on being heard if their suffering is not seen and understood	3 Patients strongly expressed their suffering and deep worries about wanting to be seen and understood	4 Patients experienced having their suffering seen and understood
Sub‐themes	Taking initiative and making contact	Being present and advocating oneself	Expressing desire to get the needed care and suffering alleviated	Reaching reciprocity and equality
	Greeting, talking and discussing in a friendly way	Being brave and demanding to be heard	Inviting in serious situations by appealing to suffering	Building a mutual caring relationship
	Exploring the atmosphere for proposing things correctly			

### 3.1 Patients Tried to Contact Healthcare Persons in a Human‐To‐Human Way With Polite Small Talk and Discussion

This theme expresses how much the participants paid attention to making a good first impression. The participants tried to express their deepest worries about their health problems and suffering by *taking the initiative and making contact*.

The participants were aware that, as patients, their responsibility and initiative are principally needed to create a caring relationship. The participants made contact first, mostly by telephone or digital communication—recently more common—or by starting a face‐to‐face discussion during care.

*It is so that initiative is more needed; I mean a patient's initiative. To create the [caring] relationship, I will make the contact*.

One participant recognised the patient's role as an inviting component: *‘I try very carefully to tell them … and to be an actor, an inviter’*.

Another said, *‘Without my own initiative, I would be quite alone*’.

Participants described ordinary ways to invite healthcare persons into a caring relationship, such as *greeting, talking, and discussing in a friendly way*. Participants were aware of the impact of their own polite and friendly behaviour and talked about the atmosphere: *‘I try to say something nice … to lighten the atmosphere by myself’*. They created normal human‐to‐human give‐and‐take situations and mentioned personal matters in the discussions: *‘I have it [conversation] also to the personal level, but not too personal. I say, for example, that we have not met before, and have you been working here for a long time … to show some interest …’*.

One participant was more courageous and initially used humour: *‘I use humour … it may become … easier if you a little have humour about yourself—something in me or what I have done …’*.

Human‐to‐human communication was about *exploring the atmosphere and proposing things correctly*. Participants in an ordinary discussion with healthcare persons knew about *the right timing* for different questions and themes: *‘… if I see the nurse wants to talk; I read the person if I can …’* Participants communicated nonverbally in a human‐to‐human relationship with the nurses regarding their heavy, important work by paying attention to them. Participants examined healthcare persons' nonverbal behaviour to determine how inviting they could be and whether a response would be generated.

*“I think and weigh, can I ask … I have been scanning how busy they seem and how often they visit beside my bed. Are they visiting spontaneously, are they just walking by and checking that way, or are they visiting here near …”*

The participants planned how to present the history of their illness and their needs in the care plan. A patient's use of pleasant and accurate expressions helped that patient and the healthcare persons; one participant called it *‘a hospital game’*. The participants regulated their initiative so it was appropriate for the situation because they respected the healthcare persons, thus building confidence.

Sometimes the participants received no response to their invitations.

*You try to talk or say something, and ‘hmm’, they say—and don't answer anything more than that*.

The participants developed a more noticeable invitation after this kind of experience.

### 3.2 Patients Advocated for Themselves and Insist on Being Heard if Their Suffering Is Not Seen and Understood

The participants' invitation to announce their suffering was stronger in this theme. The participants were observant when meeting the healthcare person and used a louder voice—verbally and non‐verbally. These participants put aside the polite approach that Theme 1 presents when necessary.

*Being present* was nonverbal and not always an easy way for participants to show their interest and draw the healthcare persons' attention. Being present included attentiveness and an ability to continue the discussion: *‘… you should be, how to say it … present and awake, if you are demanding but not getting the answer … or not getting attention … this is the point when you must act … but it is not necessarily so easy … because emotions are on the surface …’* One participant saw that a patient's presence is most important in the beginning: *‘In the first encounter when the nurse comes … when you go into the situation … are you present … it has an effect’*.

The participants had the opportunity *to advocate for themselves*: *‘I have learnt to use my voice and explain things’*. The participants had many contacts with healthcare persons in the emergency room, where their diseases were not well known. Learning how to lead the conversation towards the proper focus was crucial. One participant was happy about being able to influence the physician: *‘I was able to influence him [doctor] so much that he believed in what I said; he changed his opinion’*.

Making one's suffering heard required the courage to be *demanding and brave*. Most participants understood that healthcare persons are not mind readers; hence, being demanding was normal and necessary: *‘When you call the healthcare person, you must be brave in a kind way and be able to make demands’*.

### 3.3 Patients Strongly Expressed Their Suffering and Deep Worries About Wanting to Be Seen and Understood

The participants inviting healthcare persons forgot polite behaviour (Theme 1) and appropriate demanding (Theme 2) when an urgent need existed or the suffering remained unmet by healthcare persons. Their voices became fearful and sometimes angry or desperate. They were ready to instruct what should be done next concerning their care.

One participant advised that a patient *‘should offer oneself into the caring relationship’* because she experienced that *‘… nurses are not always so inclusive … It [care] almost does not begin until the patient by herself should … if she wants to achieve something. Maybe I am pretty harsh in my estimation, but it is my experience’*. The participant experienced that patients must be extremely determined to *express the desire to get the needed care and their suffering alleviated*: *‘I told [nurses] that I felt terribly sick and bad’*.

Participants were not shy in pressing the nursing call button, ignored the professional's emergencies, and were unapologetic about their needs: *‘… One night, a nurse came to tell me “not to press the alarm in vain because there are just two of us” … I could not turn in the bed … oh my God, I became angry … and said, “I will press the nursing call as much as I need”’*.

One participant feared losing her life if the physician did not know or understand her situation: *‘You must be brave and defend yourself … that you'll lose your life if … the new doctor … doesn't really understand …*’ One participant understood his own *serious situation and appealed to his suffering* to motivate the physician: *‘If you cannot send me into the special health care, I'll die* … *I had to rush[to]the doctor’*.

Participants were angry and tired of continuous suffering; one purposely stepped into the professionals' area to give instructions. Participants were aware that patients must be resilient in maintaining contact in their care: *‘One must develop [a] tough skin; if you want to stay alive, you must always tell it [needs and problems] again and again’*.

The participants still sought a human‐to‐human relationship despite expressing anger and tiredness: *‘I said sometimes too rudely … then I apologised; let's start from the beginning’*.

Some factors influenced participants' invitations. Healthcare authority in medicine and nursing was considered a prevailing restricting matter when invited.

*… there is a gap; there are experts and professionals, and there is a patient. This gap is very strong in medicine; some have the knowledge … and some depend on that knowledge. They are put into the position of receiving from them who have the knowledge*.

Another influencing factor was a participant's self‐respect, which could improve the care and help navigate the authority to behave more demandingly: *‘When you have self‐respect, you can take your place despite having less power’*.

An initially inviting participant gained self‐respect and vice versa: *‘… if your self‐respect improves, then being the actor and the inviting one is easier’*.

One participant protected his dignity by not being demanding because demanding resembles begging: *‘If I demand and demand and then I somehow beg … it is a negative thing and decreases my dignity’*. The participant kept his invitation at the polite human‐to‐human level; such an invitation might sometimes be imperceptible to healthcare persons.

Participants also had negative experiences with issuing strong invitations and not paying enough attention to the right moment and situation: *‘If you shout too loud, the door will be thrown closed, and nobody listens anymore’*.

### 3.4 Patients Experienced Having Their Suffering Seen and Understood

Gaining involvement in their care was meaningful to the participants. *Reciprocity* means that participants could be *equal* to the healthcare persons—sharing their own suffering, having their suffering understood and seen, and having some responsibility in the caring relationship.

One participant wanted the healthcare person to notice her expertise: *‘It is interaction, and maybe there is some kind of equality … my attendance is more permissible and wanted, and you can take it that it is I who is the expert of my own situation’*.

Participants expressed relief and having confidence in the healthcare persons if their suffering and troubles were seen, and a *mutually caring relationship* was reached. Mutual talking, listening and discussion about the care led to good quality care: *‘When you arrive at that with the doctor or nurses—that you both listen and weigh options—that is good care’*.

## 4 Discussion

This study aimed to explore patients' experiences in inviting healthcare persons into caring relationships. The findings highlight that patients invited actively, and their invitation reflected their most important need: to have healthcare persons see and understand their suffering, which can be interpreted as an overall theme.

The findings showed that patients were not passively awaiting healthcare persons' invitations; rather, when creating the caring relationship, they initially invited in a human‐to‐human way via conventional talking and taking responsibility for the Other, as Lévinas [23, 24, 25] explained regarding how human relations are built. It is ethically important for healthcare persons to listen to a patient's invitation [21]—even if it is just a polite greeting—because such invitation carries underlying messages of suffering [20].

Patients' human‐to‐human invitations included friendliness, humour, a lot of talk, communicating their respect to the professionals, and exploring the atmosphere to clarify their possibilities of inviting and determining whether the invitation would be received. This can be interpreted as an expression of patients' vulnerability and voluntariness [12] when they gave healthcare persons plenty of room to invite or not invite. This can also be interpreted as a patient's continuous appeal to be invited into a caring relationship [19].

The findings illuminated the patients' desire for a caring relationship motivated by their suffering. They strived to build relationships in responsibility [23], reciprocity [17] and mutuality [21], which can be interpreted that patients aimed for healthcare persons' engagement with the patients' suffering [7]. They noticed the relationship is asymmetrical because healthcare persons held professional power that can be used for a patient's benefit through knowledge and good acts [34]. The care was good quality, patients' questions were answered, and the suffering was shared, cf. [13, 14] and confirmed [15] in this kind of relationship.

The findings showed that patients flexibly used increasing activity to express their invitations, depending on whether they experienced their initial invitations being received, and this phenomenon has been sparsely discussed in caring and nursing research. Patients could advocate for themselves and insist on being heard, raising an ethical question: Are healthcare persons sometimes exceptionally hard of hearing? Although self‐advocacy, which is often encouraged by healthcare persons to empower patients, is generally seen as positive [35], it becomes ethically problematic when these healthcare persons fail to listen to vulnerable patients, unintentionally compelling them to struggle for attention and care. However, patients' ability to invite in a more intensive way possibly could generate patient‐centred communication in the care [1], achieving finally better interaction and quality of care, cf. [3, 4, 5], although the space was not given to it from the beginning.

Patients who express their readiness to receive information through invitation should be heard, and this is in line with the SPIKES protocol [9, 10]. Patients could head towards expressing their suffering clearly; then the invitations—which should be accepted, too—were very strong but might contain discourteous features directed towards the healthcare persons.

The findings showed that a patient's self‐respect and ability to navigate under a professional's authority could grow when a patient has gained experience in the care relationship; this can be interpreted as a caring relationship with mutuality, which has a strong, positive influence as growing caring [21]. A patient's ability to make contact and a patient's self‐respect were intertwined.

An alarming finding is that sometimes a patient could not make a demand because demanding decreased that patient's dignity. Thus, patients' invitations might remain invisible, cf. [17, 36] if the healthcare persons could not recognise a simple, polite call. Patients sometimes did not get their invitations through, although they used all invitation activity levels, causing sadness, anger and suffering related to the care, including feelings of worthlessness. Sometimes patients cannot express their invitation except through imperceptible non‐verbal movements, as happened in the critical care context, and the nurses did not notice them [11], and in this kind of situation a patient's invitation may appear as an appeal for charity [16]. Healthcare persons' busyness is an important reason for neglecting their patients [14], and not noticing their invitations, or even for being angry at and annoyed with them [11, 37].

In this study patients' deepest suffering was interpreted as the main substance and motive of their invitations, cf. [19]. Patients communicated their suffering to the healthcare persons and simultaneously pondered how healthcare persons reflected on their invitations, cf. [20]. A patient's invitation can be metaphorically interpreted as a diverse, effective, flexible and living riverbed for a patient's message of suffering.

## 5 Conclusion

Patients invite healthcare persons into a caring relationship to have their suffering seen and understood. Thus, a patient's invitation is part of building an equal and mutually beneficial caring relationship with healthcare persons.

Patients use different types of invitations from polite greeting to strongly expressed suffering depending on whether they experience the initial invitation as having been heard and confirmed—perhaps because the professionals cannot sense the initial human‐to‐human invitation, and that is ethically unsustainable.

The implications for clinical practice are to encourage healthcare persons to respond enthusiastically and with engagement to all kinds of patient invitations—correct, quiet and even silent—because some patients are too timid or ethically sensitive to make a demand. Nursing and medical schools should also highlight these issues. The findings may assistant hospitals improve patient interactions.

More research is needed to understand the multifaceted nature of invitations, including how patients invite in different health and life situations. For example, inpatients may provide different descriptions, and healthcare persons may perceive the patients' and their own invitations in ways we do not yet know. Additional research is needed into patients' experiences of how healthcare persons present their invitations to patients and how these invitations affect patients' dignity.

## 6 Methodological Considerations

The researchers were aware of the possibility that participants might have acted differently than they described in the interviews. Data collection through videorecording or observations could have given a new insight in the research area. Participants did not review the data or the findings as the dialogical interviews were intentionally planned to be deep and comprehensive. No repeat interviews were conducted. The researchers' theoretical and clinical backgrounds have an influence on how the text speaks to them and what kind of meaning patterns they found in the analysis [27]. The study's findings can be utilised in adult outpatient care; however, the transferability might be limited by the Finnish context.

## Author Contributions

M.L.P., A.K. and H.V.‐R. conducted the conceptualisation, planned aim, methodology, data collection, interview questions and analysis. L.F. participated in the conceptualisation, aim and analysis. M.L.P. carried out data curation and interviews, conducted initial coding and analysis, planned tables and wrote the original draft. A.K., L.F. and H.V.‐R. supervised the review and editing of the manuscript.

## Funding

The authors have nothing to report.

## Ethics Statement

The research permit (HUS/218/2022) was obtained in October 2022. HUS Ethics Committee gave the statement (HUS/13407/2022).

## Conflicts of Interest

The authors declare no conflicts of interest.

## Data Availability

The data collected and analyzed for this study are not publicly available due to ethical and legal considerations. The transcribed data are in Finnish and can be made available by special request.

## References

[1] J. Höglander , I. K. Holmström , A. Lövenmark , S. Van Dulmen , H. Eide , and A. J. Sundler , “Registered Nurse–Patient Communications Research: An Integrative Review for Future Directions in Nursing Research,” JAN 79, no. 2 (2022): 539–562, 10.1111/jan.15548. 36534429

[2] W. A. Langewitz , P. Eich , A. Kiss , and B. Wössmer , “Improving Communication Skills—A Randomized Controlled Behaviorally Oriented Intervention Study for Residents in Internal Medicine,” Psychosomatic Medicine 60, no. 3 (1998): 268–276, 10.1097/00006842-199805000-00009. 9625213

[3] D. L. Stone and E. P. Wempe , “A Review of Patient‐Centered Care in Interventional Oncology Settings,” CJON 29, no. 6 (2025): E40–E44, 10.1188/25.CJON.S1.E40-E44. PMC12919335 41269740

[4] J. Chu , N. Wang , Y. S. Choi , and D. H. Roby , “The Effect of Patient‐Centered Communications and Racial Concordant Care on Care Satisfaction Among U.S. Immigrants,” Medical Care Research and Review 78, no. 4 (2019): 404–412, 10.1177/1077558719890988. 31793385

[5] D. Shin , Y. Lee , E. Song , J. Kim , and R. Song , “Patient‐Centred Communication as a Mediator of Nurses' Safety‐Care Activities,” Scandinavian Journal of Caring Sciences 39, no. 3 (2025): e70103, 10.1111/scs.70103. 40887713 PMC12399778

[6] L. Del Piccolo , A. Finset , A. V. Mellblom , et al., “Verona Coding Definitions of Emotional Sequences/VR‐CoDES: Conceptual Framework and Future Directions,” Patient Education and Counseling 100, no. 12 (2017): 2303–2311, 10.1016/j.pec.2017.06.026. 28673489

[7] K. Kuchinad , J. R. Park , D. Han , S. Saha , R. Moore , and M. C. Beach , “Which Clinician Responses to Emotion Are Associated With More Positive Patient Experiences of Communication?,” Patient Education and Counseling 124 (2024): 108241, 10.1016/j.pec.2024.108241. 38537316

[8] A. K. Morgans and L. Schapira , “Confronting Therapeutic Failure: A Conversation Guide,” Oncologist 20, no. 8 (2015): 946–951, 10.1634/theoncologist.2015-0050. 26099747 PMC4524768

[9] O. Ozyemisci‐Taskiran , O. Coskun , I. I. Budakoglu , and N. Demirsoy , “Breaking Bad News in Spinal Cord Injury; a Qualitative Study Assessing the Perspective of Spinal Cord Injury Survivors in Turkey,” Journal of Spinal Cord Medicine 41, no. 3 (2018): 347–354, 10.1080/10790268.2017.1311463. 28387153 PMC6055950

[10] P. Marschollek , K. Bąkowska , W. Bąkowski , K. Marschollek , and R. Tarkowski , “Oncologists and Breaking Bad News—From the Informed Patients' Point of View. The Evaluation of the SPIKES Protocol Implementation,” Journal of Cancer Education 34, no. 2 (2019): 375–380, 10.1007/s13187-017-1315-3. 29399734

[11] K. Knutsen , R. Solbakken , and B. Normann , “The Diverse Invitations to Participate in Early Rehabilitations—A Qualitative Study of Nurse—Patient Interactions in the Intensive Care Unit,” Intensive & Critical Care Nursing 80 (2024): 103556, 10.1016/j.iccn.2023.103556. 37793317

[12] J. Petticrew , “Intensive Nursing Care: The Ministry of Presence,” Critical Care Nursing Clinics of North America 2, no. 3 (1990): 503–508, 10.1016/S0899-5885(18)30810-4. 2264975

[13] T. N. Silva , “Paterson and Zderad's Humanistic Theory: Entering the Between Through Being When Called Upon,” Nursing Science Quarterly 26, no. 2 (2013): 132–135, 10.1177/0894318413477209. 23575489

[14] K. Eriksson , The Suffering Human Being (Nordic Studies Press, 2006).

[15] K. Eriksson , Mot en Caritativ Vårdetik [Toward a Caritative Caring Ethics], ed. K. Eriksson (Åbo Akademi, 1995), 1–39. Reports from the Department of Caring Science 5/1995.

[16] U. Å. Lindström , N. L. Lindholm , and J. E. Zetterlund , “Theory of Caritative Caring,” in Nursing Theorists and Their Work, 9th ed., ed. M. Raile Alligood (Elsevier, 2018), 140–163.

[17] A. Rehnsfledt , “Mötet Med Patienten i Ett Livsavgörande Skeende [The Encounter With the Patient in a Life‐Changing Process” (Doctoral thesis, Åbo Akademi, 1999).

[18] I. Bergbom , D. Nåden , and L. Nyström , “Katie Eriksson's Caring Theories. Part 1. The Caritative Caring Theory, the Multidimensional Health Theory and the Theory of Human Suffering,” Scandinavian Journal of Caring Sciences 36, no. 3 (2021): 782–790, 10.1111/scs.13036. 34609017

[19] T. Nordman , “Människan Som Patient i en Vårdande Kultur [Human Being as a Patient in the Caring Culture” (Doctoral thesis, Åbo Akademi, 2006).

[20] L. Fagerström , “The Patient's Perceived Caring Needs as a Message of Suffering,” Journal of Advanced Nursing 28, no. 5 (1998): 978–987, 10.1046/j.1365-2648.1998.00822.x. 9840869

[21] M. J. Purnell , A. Boykin , and S. O. Schoenhofer , “The Theory of Nursing as Caring: A Model for Transforming Practice,” in Nursing Theorists and Their Work, 9th ed., ed. M. Raile Alligood (Elsevier, 2018), 293–308.

[22] A. Boykin and S. O. Schoenhofer , “Nursing as Caring. A Model for Transforming Practice,” (The Project Gutenberg Ebook of Nursing as Caring, 2026, https://nursology.net/wp‐content/uploads/2018/09/nursing‐as‐caring‐pdf.pdf. 8367273

[23] E. Lévinas , Totality and Infinity, an Essay on Exteriority (Martinus Nijhoff Publishers, 1979).

[24] L. Rosmarin , “The I‐You Relationship in the Works of Emmanuel Lévinas,” Angelaki 6, no. 2 (2001): 7–14, 10.1080/09697250120076358.

[25] P. Kemp , Emmanuel Lévinas—En Introduktion (Daidalos, 1992).

[26] S. Brinkmann and S. Kvale , Interviews. Learning the Craft of Qualitative Research Interviewing, 3rd ed. (Sage, 2015).

[27] V. Braun and V. Clarke , “One Size Fits All? What Counts as Quality Practice in (Reflexive) Thematic Analysis?,” Qualitative Research in Psychology 18, no. 3 (2020): 328–352, 10.1080/14780887.2020.1769238.

[28] L. A. Palinkas , S. M. Horwitz , C. A. Green , J. P. Wisdom , N. Duan , and K. Hoagwood , “Purposeful Sampling for Qualitative Data Collection and Analysis in Mixed Method Implementation Research,” Administration and Policy in Mental Health 42, no. 5 (2015): 533–544, 10.1007/s10488-013-0528-y. 24193818 PMC4012002

[29] M. M. Hennink , B. N. Kaiser , and V. C. Marconi , “Code Saturation Versus Meaning Saturation: How Many Interviews Are Enough?,” Qualitative Health Research 27, no. 4 (2016): 591–608, 10.1177/1049732316665344. 27670770 PMC9359070

[30] M. Vaismoradi , H. Turunen , and T. Bondas , “Content Analysis and Thematic Analysis: Implications for Conducting a Qualitative Descriptive Study,” Nursing & Health Sciences 15, no. 3 (2013): 398–405, 10.1111/nhs.12048. 23480423

[31] V. Braun and V. Clarke , “Using Thematic Analysis in Psychology,” Qualitative Research in Psychology 3, no. 2 (2006): 77–101, 10.1191/1478088706qp0630a.

[32] A. Tong , P. Sainsbury , and J. Craig , “Consolidated Criteria for Reporting Qualitative Research (COREQ): A 32‐Item Checklist for Interviews and Focus Groups,” International Journal for Quality in Health Care 19, no. 6 (2007): 349–357, 10.1093/intqhc/mzm042. 17872937

[33] Finnish National Board on Research Integrity TENK , The Finnish Code of Conduct for Research Integrity and Procedures for Handling Alleged Violations of Research Integrity in Finland (Finnish National Board on Research Integrity TENK, 2026), https://tenk.fi/sites/default/files/2023‐11/RI_Guidelines_2023.pdf.

[34] I. Hynnekleiv , J. K. Jensen , T. Giske , H. Lausund , E. Mæland , and K. Heggdal , “Patients' and Nurses' Experiences of Caring in Nursing: An Integrative Literature Review Across Clinical Practices,” Journal of Clinical Nursing 33, no. 4 (2023): 1233–1255, 10.1111/jocn.16964. 38093547

[35] T. L. Hagan and E. Medberry , “Patient Education vs. Patient Experiences of Self‐Advocacy: Changing the Discourse to Support Cancer Survivors,” Journal of Cancer Education 31, no. 2 (2016): 375–381, 10.1007/s13187-015-0828-x. 25846573 PMC4598253

[36] M. Arman , A. Rehnsfeldt , L. Lindholm , E. Hamrin , and K. Eriksson , “Suffering Related to Health Care: A Study of Breast Cancer Patients' Experiences,” International Journal of Nursing Practice 10, no. 6 (2004): 248–256, 10.1111/j.1440-172x.2004.00491.x. 15544580

[37] L. Govasli and B. A. Solvoll , “Nurses' Experiences of Busyness in Their Daily Work,” Nursing Inquiry 27, no. 3 (2020): e12350, 10.1111/nin.12350. 32133740

